# Supplementary material for: SyNDI: synchronous network data integration framework
Source: BMC Bioinformatics. 2018 Nov 6;19:403. doi: 10.1186/s12859-018-2426-5 (PMC6219086; doi:10.1186/s12859-018-2426-5)
Supplement: Supplementary file 5 — Files of the example presented in Additional file 4. This additional file is a zip package that contains all files used in Additional file 4. Detailed descriptions of each of these files are presented in a README file included in this zip package. (GZ 30411 kb) [file 12859_2018_2426_MOESM5_ESM.gz › SyNDITools/Meme2Fimo/meme_4.9.0_4.tar.gz!/meme_4.9.0/doc/fasta-io.xml]

fasta-io


# fasta-io

**Usage:** `fasta-io [options] <sequences>`

**Description:**

This utility reads a sequence file in FASTA format and writes
it to standard out.

**Input:**

- <*sequences*> - a file containing a set of sequences in
  FASTA format.

**Output:**

The program prints to standard output.

**Options:**


**Bugs:** None known.
